# σ_2_ Receptor and Its Role in Cancer with Focus on a MultiTarget Directed Ligand (MTDL) Approach

**DOI:** 10.3390/molecules26123743

**Published:** 2021-06-19

**Authors:** Francesca Serena Abatematteo, Mauro Niso, Enza Lacivita, Carmen Abate

**Affiliations:** Dipartimento di Farmacia-Scienze del Farmaco, Università degli Studi di Bari ALDO MORO, Via Orabona 4, 70125 Bari, Italy; francesca.abatematteo@uniba.it (F.S.A.); mauro.niso@uniba.it (M.N.); enza.lacivita@uniba.it (E.L.)

**Keywords:** σ receptors, σ_2_ receptor, MultiTarget Directed Ligand (MTDL), resistant cancer, collateral sensitivity

## Abstract

Sigma-2 (σ_2_) is an endoplasmic receptor identified as the Endoplasmic Reticulum (ER) transmembrane protein TMEM97. Despite its controversial identity, which was only recently solved, this protein has gained scientific interest because of its role in the proliferative status of cells; many tumor cells from different organs overexpress the σ_2_ receptor, and many σ_2_ ligands display cytotoxic actions in (resistant) cancer cells. These properties have shed light on the σ_2_ receptor as a potential druggable target to be bound/activated for the diagnosis or therapy of tumors. Additionally, diverse groups have shown how the σ_2_ receptor can be exploited for the targeted delivery of the anticancer drugs to tumors. As the cancer disease is a multifactorial pathology with multiple cell populations, a polypharmacological approach is very often needed. Instead of the simultaneous administration of different classes of drugs, the use of one molecule that interacts with diverse pharmacological targets, namely MultiTarget Directed Ligand (MTDL), is a promising and currently pursued strategy, that may overcome the pharmacokinetic problems associated with the administration of multiple molecules. This review aims to point out the progress regarding the σ_2_ ligands in the oncology field, with a focus on MTDLs directed towards σ_2_ receptors as promising weapons against (resistant) cancer diseases.

## 1. Introduction

Treatment of cancer, which is a major public health problem worldwide and the second leading cause of death (in the USA) [1], has changed a great deal over the years. The first modern therapeutic approach dates back to the end of 1800s with the discovery of X-rays. From that moment, amazing scientific and medical progresses have furnished a plethora of approaches that have led to increasingly specific and effective treatments. From the birth of chemotherapy, based on cytotoxic antitumor drugs to genetic engineering studies, which provided monoclonal antibodies, immune checkpoint inhibitors, and Chimeric Antigen Receptor T cell therapies (CAR-T), treatment of cancer has drastically changed over the years and life expectancy of people suffering from this pathology has considerably improved [2,3].

Cancer is a complicated pathology because of the many mechanisms responsible for the evasion from the regulatory circuits, which ensure a correct cell growth. Besides the enhanced angiogenesis, the most important mechanisms that sustain the progression of the pathology consist of the production of growth factors and the insensibility to anti-growth factors (which allow a limitless replicative potential); the ability to evade apoptosis and to escape from the primary tumor mass to create metastasis [4].

This plurality of mechanisms justifies the need of a polypharmacological approach to treat the pathology working on two or more targets at the same time, in order to produce synergic effects and increase the efficacy of the treatment. Multifunctional therapies can be based either on the well validated use of combinations of drugs administered together, or on the use of a single multitarget directed ligand (MTDL), whose interaction with different targets exerts more pharmacological effects. Despite the therapeutic success, the main limitation of the former approach lies in the diverse pharmacokinetic and metabolic profiles of the drugs that may lead to multiple toxic metabolites and side effects, compared to a single drug administration. Thus, the MTDL approach is attracting interest as a strategy to be exploited to treat cancer and the other pathologies based on different factors.

Both the Sigma (σ) receptor subtypes, σ_1_ and σ_2_, are involved in cancer disease and, have been often exploited as targets for the development of MTDLs to synergize with the antitumor action mediated by other targets. In this review, we only briefly discussed about the σ receptor, while we focused more on the σ subtype and the structural insights of the σ-directed MTLDs in the context of cancer.

## 2. σ Receptors

σ proteins, which were thought to belong to the opioid receptor family until 1976, were later identified as an independent class of receptors divided into two different subtypes [5]. The σ_1_ subtype was cloned in the early 1990s, and its crystal structure was recently disclosed [6], while the σ_2_ receptor was only lately identified as the TMEM97 protein [7] and its crystal structure has been resolved during the preparation of this review [8]. Although the mechanisms of action of the two proteins need to be fully elucidated, they both appear as intriguing targets for the development of therapies useful for a wide range of pathologies [9,10].

### 2.1. σ_1_ Receptor

σ_1_ receptor is a 223-amino-acid protein characterized by a high level of similarity with the ERG2p, a C8-C7 sterol isomerase expressed in yeast, even if no isomerase activity has been attributed to σ_1_ receptors. The protein structure consists of five α-helices and ten β-strands. The N-terminus crosses the Endoplasmic Reticulum (ER) membrane and protrudes into the lumen forming a transmembrane domain (TMD), while the flat and hydrophobic C-terminus is associated to the cytosolic surface of the ER. The σ_1_ protein is mainly localized at the interface between mitochondria and ER, a space commonly named mitochondrial associated endoplasmic reticulum membrane (MAM), where it interacts with proteins to modulate Ca^2+^ fluxes between ER and mitochondria [11,12]. This receptor has been lately defined as a ‘pluripotent chaperone’ that interacts with several client proteins modulating their activity [10]. One of its most studied interactions is with the binding immunoglobulin proteins (BiP), with which it is associated in rest conditions. Upon stress (e.g., Ca^2+^ depletion from ER), the receptor dissociates from BiP and chaperones the Inositol 1,4,5-triphospate receptor type 3 (IP3R3) at MAM, increasing the Ca^2+^ flow from ER to mitochondria to guarantee cell energy needs [11].

σ_1_ receptor’s role in neurodegenerative diseases has been extensively studied and reviewed, and its therapeutic exploitability as druggable target is witnessed by the ligands in clinical trials for treating pathologies such as Alzheimer disease (AD), Huntington disease (HD) etc [13,14]. Less explored, but still intriguing under the therapeutic perspective, is the σ_1_ receptor role in cancer. The σ_1_ receptor exploitation as a target for the development of MTDLs addressing the diverse pathologies in which this σ subtype is involved has been recently reviewed [15].

#### 2.1.1. σ_1_ Receptors Involvement in Cancer 

High levels of σ_1_ receptors have been found in human cancer cell lines of breast, lung, prostate, colon, melanoma, CNS, kidney and pancreas [16]. However, despite controversial results in breast adenocarcinoma [17,18], Xu reported significant correlation between σ_1_ receptor expression and progression of esophageal squamous cell carcinoma [19] and hilar cholangiocarcinoma [20].

σ_1_ receptor density was also found to be increased in breast cancer cells with high metastatic potential, supporting a direct correlation between the expression of the receptor and the aggressiveness of the pathology [21].

Moreover, scintigraphy studies performed using (*N*-[2-(1′-piperidinyl)ethyl]-3-^123^I-iodo-4-methoxybenzamide), a selective ligand for the σ_1_ receptor, on patients with primary breast cancer revealed that the ligand was specifically retained in diseased tissues rather than healthy ones [22].

Finally, as reviewed by Kim, both σ_1_ receptor claimed agonists (i.e., (+)-SKF10047, PRE-084 and (+)-pentazocine) and antagonists (i.e., haloperidol, SR31747A, Rimcazole and BD1047) demonstrated anticancer activities [23]. This data, that may raise doubts about the σ_1_ receptor mediated cytotoxicity, also emphasizes the likely inconvenience of the word ‘agonist’ and ‘antagonist’ in the σ_1_ receptor context, as reported by Yano et al. [24].

#### 2.1.2. σ_1_/σ_2_ Receptors MTDLs

The still-unclear involvement of the σ_1_ receptor in cancer justifies the small number of studies performed for the synthesis of σ_1_ directed MTDLs with anticancer activity. However, ligands with unintentional affinity for both receptors have been found to produce interesting antiproliferative actions.

**Figure 1:**
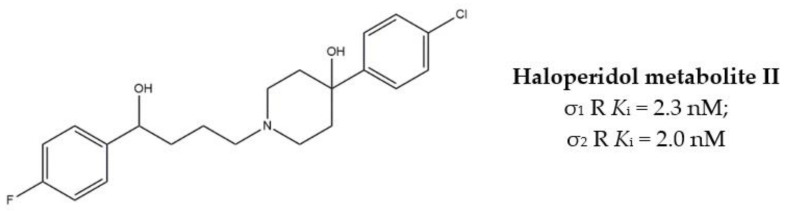
Haloperidol metabolite II with *K*_i_ values for σ receptors.

The anticancer effect produced by dual σ receptor ligands was explored by Marrazzo and colleagues, who believed that agonist activity on σ_1_ receptor and antagonist activity on σ_2_ receptor may synergize to produce an increased anticancer effect. This pharmacological profile was found in haloperidol metabolite II (HP II) (Figure 1), which was also found to be able to increment intracellular-free calcium [Ca^2+^]_i_ levels and induce apoptosis [25]. Even if the antiproliferative activity produced by HP II is modest, this compound is the first of a new class of compounds denominated ‘pan-SR ligands class’ [26,27].

With the aim to extend the pharmacological profile, HP II was conjugated with the Histone Deacetylase (HDAC) inhibitor 4-phenylbutyric acid to produce the ester derivative (*R*,*S*)-MRJF4. Despite the higher *K*_i_ values for σ receptors (with a slight preference for the σ_2_ subtype), the MTDL showed a more potent antiproliferative effect in LNCaP and PC3 prostate cancer cells than HP II, administered alone or in co-administration with 4-phenylbutyric acid. 

**Figure 2:**
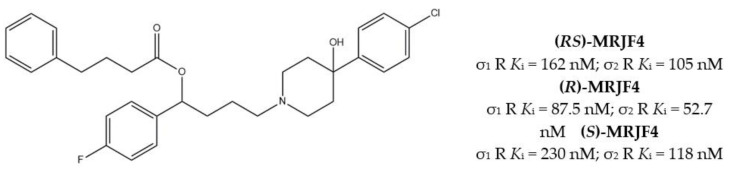
(*RS*)-, (*R*)- and (*S*)-MRJF4 and *K*_i_ values for σ receptors.

Subsequently, pure enantiomers were also tested and (*R*)-enantiomer displayed better anticancer activity than racemic mixture, probably because of its lower *K*_i_ values for both σ receptors [28] (Figure 2).

Another step in this direction was taken by Riganas and co-workers, who developed (1-adamantyl)diarylalkylamines in which the adamantyl moiety was introduced with the aim to produce activity at Na^+^ channels, whose involvement in cancer has been proven [29,30].

**Figure 3:**
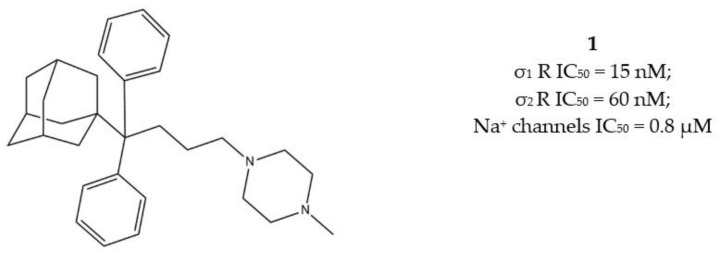
Compound **1** and its IC_50_ values for σ receptors and Na^+^ channels.

Compounds characterized by a butyl chain between the (1-adamantyl)diaryl portion and the amine function, displayed the best profile because of a valuable σ_1_ receptor antagonist activity, a weak σ_2_ receptor agonist activity and micromolar affinity for the site 2 of Na^+^ channels. Overall, piperazine derivative 1 (Figure 3) exerted the best cytotoxic effect on ovarian cancer cells (IGROV-1) and a good in vitro antiangiogenic activity on normal cell lines such as the Human Umbilical Vein Endothelial Cells (HUVEC).

Taking into consideration the association of the cytotoxic effect together with the analgesic effect against neuropathic pain obtained by the block of Na^+^ channels, compound **1** appeared to be endowed with a good therapeutic profile in the treatment of cancer. These few examples show how the σ_1_ receptor-based development of MTLDs is also a promising approach in the treatment of cancer.

### 2.2. σ_2_ Receptor

σ_2_ receptor was first identified by Hellewell and colleagues who discovered that [^3^H]-DTG was able to specifically bind two proteins in rat liver: a 25 kDa and a 21.5 kDa protein. Dextrallorphan (DXA) was able to block the binding of the radioligand with the 25 kDa protein (i.e., σ_1_ receptor), but was unable to block the binding with the 21.5 kD protein, which was named σ_2_ receptor [5].

In 2006, upon an affinity chromatography purification performed on a lysate from the σ_2_ overexpressing SK-N-SH neuroblastoma cells, Colabufo et al. advanced the hypothesis of the σ_2_ receptor identification with histone proteins. Alternatively, the subnanomolar affinity σ_2_ receptor ligand PB28, whose amino-derivative was used for the functionalization of the chromatographic column, could bind the histones (i.e., H3.3A, H2B, H2A.5 and H2.1) which were identified by MALDI-MS and LC-MS-MS [31].

To support this hypothesis, the interaction between PB28 and the histone proteins was evaluated by computational approaches (homology model and docking) that highlighted two specific high-affinity binding sites for PB28 on the H2A/H2B histone dimer. Experimental supports were provided by: (*i*) the verified nanomolar affinity binding of [^3^H]PB28 [32] with the reconstituted human histone dimer H2A/H2B, and (*ii*) the higher concentration of [^3^H]PB28 in the nuclei rather than in the cytosol of cancer cells. These data suggested that the effect of PB28 may be exerted through the interaction with nuclear proteins [33]. Nevertheless, studies with σ_2_ receptor fluorescent ligands did not show nuclear localization [34,35,36], and a new hypothesis about the σ_2_ receptor identity emerged. Xu and colleagues proposed that the σ_2_ receptor binding site belonged to the progesterone receptor membrane component 1 (PGRMC1) complex. The study was based on two important pieces of evidence: (*i*) the fluorescein-azido-derivative WC-21, a σ_2_ receptor fluorescent ligand, was able to irreversibly bind PGRMC1 in rat liver; (*ii*) the nitrobenzofurazan-carbamate derivative SW120 colocalized with PGRMC1 and with molecular markers of ER and mitochondria in HeLa cells. The hypothesis was also validated by radioligand binding experiments performed on overexpressing and knockdown PGRMC1 cell lines [37]. On this basis, subsequent studies treated the σ_2_ receptor and PGRMC1 as the same entity despite some inconsistencies, and pharmacological tools useful to detect PGRMC1 mediated activity were used to define the pharmacological action of σ_2_ receptor ligands. 

The matter about the identity of the σ_2_ receptor was reopened few years later, when two separate groups conducted independent experiments that led to the same conclusion. Chu and colleagues knocked-out (KO) or overexpressed PGRMC1 in mouse motor neuron cell lines (NSC34). Binding studies with [^3^H]-DTG and photolabeling studies with [^125^I]-IAF provided similar results in all cells: wild type, PGRMC1-KO and overexpressing PRGMC1. Moreover, affinities of DTG and haloperidol for PGRMC1, were found to be three orders of magnitude lower than the values reported for the σ_2_ receptor [38].

At the same time, through a combination of Western blot and radioligand binding assays, Pati et al. demonstrated that the overexpression or knock-down (KD) of PGRMC1 in MCF7 cells (widely used as a model for σ_2_ receptor activity) did not change the density of σ_2_ receptors. These results were corroborated by flow cytometry and confocal microscopy experiments in the same cells [39]. The σ_2_ receptor fluorescent ligands (F412, NO1 and PB385) [34,35,40] used to mark σ_2_ receptors were displaced by PB28 and DTG, but were not displaced by the PGRMC1 inhibitor AG205 and did not colocalize with the fluorescent anti-PGRMC1 antibody. Importantly, titration calorimetry assays demonstrated that PB28 has no affinity for PGRMC1 dimer or monomer, while DTG showed only modest affinity for the dimer [39].

The last hypothesis about σ_2_ receptors identity has been proposed by Alon and colleagues in 2017, when the σ_2_ receptor was identified as the TMEM97 (also known as MAC-30), an ER resident protein involved in cholesterol homeostasis [41] and in the Niemann–Pick type C disease as NPC1-interacting protein [42].

An affinity chromatography purification of calf liver homogenate was performed, and the proteins interacting with the σ_2_ receptor piperazine ligand JVW-1625 were isolated. Binding studies using [^3^H]DTG were performed on the detected membrane proteins upon overexpression in HEK293 cells, and TMEM97 emerged as the most likely candidate. Results from several experiments led the authors to conclude that TMEM97 is synonymous of the σ_2_ receptor. In particular, (*i*) in PC-12 cells, the KD of TMEM97 produced a decrease in σ_2_ receptors expression; (*ii*) in Sf9 insect cells, modified to overexpress the human TMEM97, binding affinity of [^3^H]DTG was comparable to the values reported in the literature for σ_2_ receptor; (*iii*) σ_2_ ligands belonging to diverse chemical classes, showed binding affinities for TMEM97 consistent with their previously reported σ_2_ receptor-binding affinities; (*iv*) the *K*_i_ values of two TMEM97 ligands, Elacridar and Ro 48-8071, to Sf9 membranes overexpressing TMEM97, were identical to those measured in MCF7 (that natively overexpress σ_2_ receptors); (*v*) site-directed mutagenesis performed on Glutamate (E) and Aspartate (D) residues revealed that D29 and D56 are necessary for ligand binding, similarly to what happens in the σ_1_ receptor binding site [7].

Moreover, during the drafting of this review, the same group that identified the TMEM97 as the σ_2_ receptor, published the crystal structure of the protein in complex with roluperidone and PB28, shedding light on the most intriguing mystery of the story of this class of receptors [8].

#### 2.2.1. σ_2_ Receptor Reference Ligands

Scientific literature reports a plethora of more or less selective ligands for the σ_2_ receptor. Most of them have been recently reviewed [9] and some interesting classes are briefly described below and reported in Table 1:

- **Morphans.** They were developed in the 1990s, and emerged as the first class of selective σ_2_ receptor ligands. These compounds were obtained by insertion of a benzylidene moiety in 8-position of the morphan system that is endowed with mixed affinity for µ and σ_2_ receptors [43,44].
- **Indoles (Siramesine-like compounds).** They were developed starting from indole-3-yl-alkyl-arylpiperazines as 5-HT_1A_ agonists [45]. This class of compounds was generated through replacement of the piperazine with constrained arylpiperidines and introduction of a 4-florophenyl group at the indole N-atom leading to siramesine, that despite its subsequently reported lack of selectivity [46], is still widely used as a reference compound. Binding to phosphatidic acid [47], ROS formation [48], lysosomotropic properties [49], release of cytochrome C by mitochondria [50] were reported as mechanisms of action of siramesine, that eventually lead to tumor cells death.
- **Granatanes.** This class of ligands was developed from BIMU-1, a 5-HT_3_/5-HT_4_ serotonin receptor ligands with nanomolar affinity for σ_2_ receptors. The bicyclo-octane core of BIMU-1 was replaced by a 9-azabicyclo[3.3.1]nonane (granatane). In addition, the cyclic urea group was opened and replaced by a phenylcarbamate moiety [51,52]. Moreover, the nitrogen atom was functionalized with a benzyl group (WC26 and WC59 [53]) or ω-aminoalkyl chains (SV119 and SW43 [54]) leading to optimal σ_2_ ligands.
- **Benzamides**. Structural modifications on D_3_ receptor ligands led to σ_2_ receptor high affinity flexible and selective benzamides. The most successful compounds, i.e., RHM-1 [55] and ISO-1 [56], were also produced as radioligands [57] to perform σ_2_ receptor binding assays or clinical PET studies for the imaging of tumors [58,59,60,61]. An intramolecular H-bond, which forces the flexible benzamides in a bicyclic conformation, was postulated for the interaction of these compounds with the σ_2_ binding site. Therefore, compound 2 and analogues, in which such H-bond conformation was mimicked, were produced. The nanomolar affinities shown by the rigid bicyclic benzamides validated the hypothesis [62,63]. Importantly, high selectivity rates characterized flexible and rigid benzamides. Corresponding reverse amides were produced (rigid and flexible anilides) as well as the corresponding flexible and rigid anilines, with agreeing results [64].
- **Cyclohexyl piperazines.** This class, which is based on its lead compound PB28, was developed starting from serotoninergic arylpiperazines [65]. During the last few decades, several analogues were developed and recently reviewed, mostly with the aim of reducing the lipophilicity, as studies on [^11^C]-radiolabelled PB28 showed high nonspecific binding in mouse brain in vivo [66,67]. However, within the more polar series no ligand showed affinity values comparable to PB28 [68]. Nonetheless, interesting biological profiles in terms of selectivity (compound 3, Table 1) or cytotoxic activity in cancer cells were obtained. Intriguingly, PB28 showed promising anti-SARS-CoV-2 activity in vitro [69], although the effect was later ascribed to the induction of phospholipidosis as an off-target effect [70].

**Table 1:** Affinities for σ_1_ and σ_2_ receptors (σ_1_ R and σ_2_ R) of reference σ_2_ R ligands.

Name	Structure	σ_1_ R *K*_i_ nM	σ_2_ R *K*_i_ nM	Reference
**Morphans:**	**-**			
(+)-CB-64D	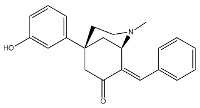	3063	16.5	[43]
(+)-CB-184	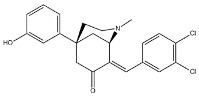	7436	13.4	[43]
**Siramesine**	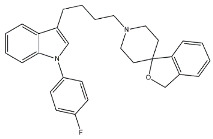	10.5	12.6	[46]
**Granatane:**				
BIMU-1	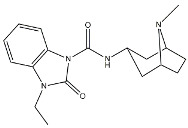	6300	32	[51]
WC26	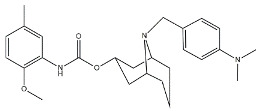	1436.5	2.58	[53]
WC59	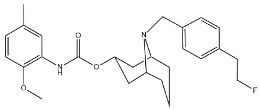	1710.5	0.82	[53]
SV119	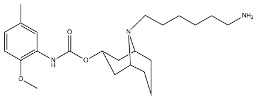	1418	5.19	[54]
SW43	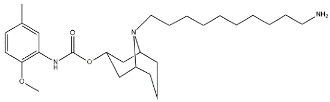	134.3	7.07	[54]
**Benzamides:**				
RHM-1	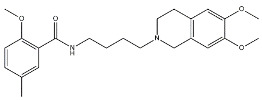	3078	10.3	[55]
ISO-1	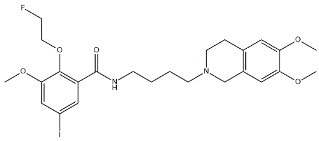	2150	0.26	[56]
2	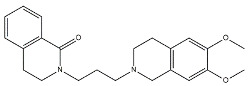	709	4.74	[62]
**Cyclohexyl piperazines:**				
PB28	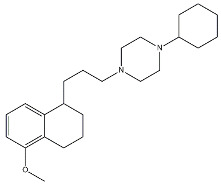	0.38	0.68	[68]
3	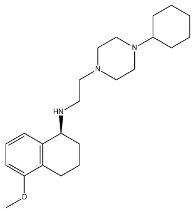	23.3	8.52	[68]

#### 2.2.2. σ_2_ Receptor Involvement in Cancer

σ_2_ receptors are associated with a well-established involvement in cell proliferation, although, recently, strong pieces of evidence have also demonstrated the involvement of this receptor in neurodegenerative diseases such as the AD [71]. Indeed, a small σ_2_ receptor modulator, named CT1812 (Elayta^R^), blocks, via the σ_2_ receptor, the binding of different Aβ oligomers to neuronal receptors and displaces them reducing Aβ-induced synaptic toxicity. Thus, Elayta has undergone clinical trials, and phase II clinical studies are still recruiting [72], highlighting how the σ_2_ protein is a promising target for cancer and CNS diseases. Many pieces of evidence demonstrate that σ_2_ receptors are able to induce cell death through different mechanisms, and σ_2_ ligands inducing cytotoxicity are identified as σ_2_ agonists. Among the effects induced by σ_2_ ligands, lysosomal dysfunction and ROS production were proved as cytotoxic mechanisms [49,73]. Additionally, the σ_2_ receptor modulates ER stress [74], likely through control of Ca^2+^ release because of direct and indirect interaction with IP3 receptor, ryanodine receptors and Sarco-Endoplasmic Reticulum Calcium ATPase (SERCA) [75]. Additionally, store-operated Ca^2+^ Entry (SOCE) downregulation [76], activation of caspase and mitochondrial superoxide production [77] and activation of p53- and caspase-independent apoptotic pathways [78] have been associated with the cytotoxicity of σ_2_ receptor ligands. Nevertheless, Mach et al. showed that the cytotoxicity of σ_2_ agonists such as PB28 and SW43, is independent from TMEM97 and PGRMC1. Indeed, KO of TMEM97, PGRMC1 or both proteins in HeLa cells, did not alter the cytotoxic effect of these ligands, whose antiproliferative action does not seem to be mediated neither by TMEM97 nor by PGRMC1 [79]. However, the effect of the two proteins on the above described cytotoxic mechanisms, in the presence of the σ_2_ ligands still needs to be investigated.

#### 2.2.3. MTDLs Based on Granatane SV119 and SW43

**Figure 4:**
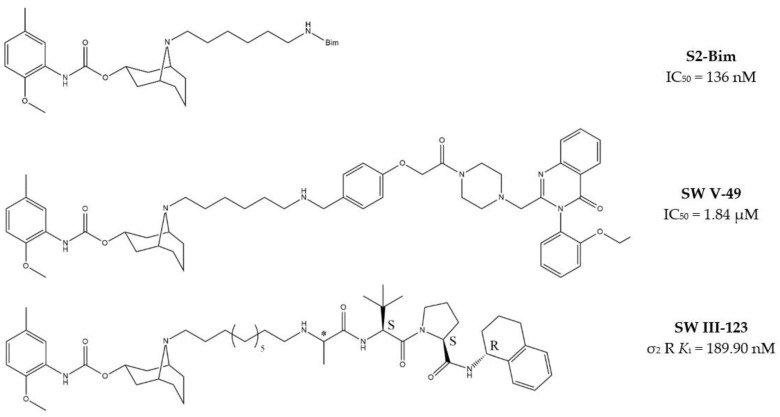
Granatane-based MTDLs: S2-Bim (peptide sequence: EIWIAQELRRIGDEFNAYYAR-OH), SW V-49 and SW III-123 structures and biological data.

Granatane derivatives are an optimal class of σ_2_ receptor ligands because of their pharmacological profile in terms of affinity and selectivity. Among all the ligands belonging to this class, analogues bearing an ω-aminoalkyl chain represent ideal tools in the synthesis of MTDLs. The easily conjugatable amino-group can be transformed into the corresponding carbamate and amide derivatives. Among these ligands, Spitzer and colleagues chose granatane derivative SV119 (Table 1), whose intrinsic cytotoxicity, affinity and selectivity for the σ_2_ receptor prompted its exploitation for the targeted delivery of the MTDL to pancreatic cancer cells. Indeed, SV119 was conjugated with cell-death inducing small molecules such as 1) the pro-apoptotic peptide Bim, (a BH3-only peptide belonging to the Bcl-2 family); 2) the carboxyl-terminal modulator protein (CTMP, a mitochondrial peptide released under apoptotic conditions that inhibits protein kinase B, Akt); 3) rapamycin—a compound with activity on phosphoinositide 3-kinase (PI3K)/Akt system. Among the three MTDLs synthesized, only the peptidomimetic chimera SV119-Bim, named S2-Bim (Figure 4), produced promising effects in in vivo models of pancreatic cancer. Thus, the intrinsic cytotoxic properties of SV119 were reinforced by activation of the Bcl-2 cell death mechanism produced by Bim. However, once the treatment was discontinued, tumor growth restarted and matched the control [80].

In the following years, granatane SV119 was also linked to Erastin, a 4-quinazolinone able to inhibit cysteine/glutamate antiporter, whose function is to keep the antioxidant glutathione homeostasis. Besides the induction of ROS accumulation, Erastin can influence the activity of mitochondrial voltage-dependent anion channels, although problems of cell uptake limit its efficacy.

With the aim to obtain an Erastin based dual drug, Ohman and colleagues, demethylated the carbon atom between the piperazine ring and the 4-quinazolinone portion in order to get rid of the chirality. The obtained demethylated-Erastin was then connected to the granatane SV119 to obtain the new MTDL named SW V-49 (Figure 4). This dual drug was characterized by a better cell uptake compared to Erastin and encouraging cytotoxicity IC_50_ values in pancreatic cancer cells. In vivo studies performed on murine models of pancreatic cancer demonstrated that SW V-49 reduces tumor size and improves survival, without side-effects that are typical of gemcitabine, the reference therapeutic for pancreatic tumor treatments [81]. Always with the aim to increase the drug delivery of peptidomimetics to cancer cells, SW43 (Table 1), another granatane derivative with a longer ω-aminoalkychain, was linked to SW IV-52s, that is a mimetic compound (SMC) of the second mitochondria-derived activator of caspase (Smac) (Figure 4). Thus, the peptidomimetic derivative SW III-123 (Figure 4) was obtained and displayed a strong cytotoxicity in all ovarian cancer cell lines studied (SKOV-3, CaOV-3 and BG-1), differently from SW IV-52s, that is active only in SKOV-3. Studies performed in SKOV-3 (EC_50_ = 4.0 µM (24 h); EC_50_ = 1.40 µM (48 h)) cells to better understand cell death mechanisms revealed that the peptidomimetic ligand SW III-123 can produce degradation of inhibitor of apoptosis proteins 1 and 2 (cIAP1 and cIAP2), accumulation of NF-κB-inducing kinase (NIK) and phosphorylation of NF-κB p65. All together, these mechanisms suggest the activation of canonical and noncanonical NF-κB pathways of death. In addition, cleavage of caspase-3, -8 and -9 was observed, and the involvement of tumor necrosis factor alpha (TNFα) in the mechanisms of cell death was demonstrated for the SMC-sensitive cells, but not for the SMC-insensitive ones [82]. While in SW III-123 the chiral centers in the proline and tetralin rings and the chirality of the carbon atom bearing the *t*-butyl group were all defined, the oxopropan-2-yl group was not, so that SW III-123 was a racemic mixture. The promising results obtained with this couple of stereoisomers led to produce the pure stereoisomeric form of SW III-123 in the L-configuration to obtain SW IV-134, which was tested in pancreatic tumor cells and provided better results than the racemate, with a ten-fold higher affinity for σ_2_ receptors (σ_2_ *K*_i_ = 22.6 nM) [83].

Thus, SW IV-134 was studied in different types of tumors, such as mouse xenograft models of ovarian cancer [84] and triple-negative breast cancer [85] providing promising results.

It is worth noticing that even if these peptidomimetics are characterized by features that are usually detrimental in terms of pharmacokinetic properties, such as high molecular weight and steric hindrance, valuable affinity values and promising preclinical data were obtained. Therefore, the granatane scaffold can be considered as an important tumor-selective delivery system with the potential to reduce the side effects produced by unselective therapies.

#### 2.2.4. Collateral Sensitivity (CS) as Multitarget Strategy to Face Cancer

Collateral sensitivity (CS) was observed for the first time by Szybalaski and Bryson in 1952 during some studies on drug-resistant cultures of Escherichia coli [86]. According to this phenomenon, cancer cells, that show resistance to classical chemotherapy, demonstrate an unusual sensitivity to other drugs. One of the main mechanisms of the resistance to drugs, also called MultiDrug Resistance (MDR), is due the overexpression of efflux pumps that reduce intracellular drug concentrations to ineffective levels [87]. Among all the efflux pumps, P-glycoprotein (P-gp), MultidrugResistance-associated Protein 1 (MRP1) and Breast Cancer Resistance Protein (BCRP), which belong to the ATP-Binding Cassette (ABC) transporters, are the main responsible of this phenomenon.

Although it may sound like a contradiction, the overexpression of the efflux pumps that is responsible of the MDR, may also account for CS. Indeed, the over-working of these transporters can: (i) activate a futile hydrolysis of ATP, increasing ROS levels; (ii) sensitize cells to changes in energy levels; (iii) produce extrusion of essential substrates for cell metabolism; (iv) perturb cell membranes [88]. Drugs able to engage these mechanisms, are able to kill MDR cells more than the non-resistant counterparts, finally leading to select the non-resistant cell population, which can ultimately be treated with the conventional chemotherapeutic.

**Figure 5:**
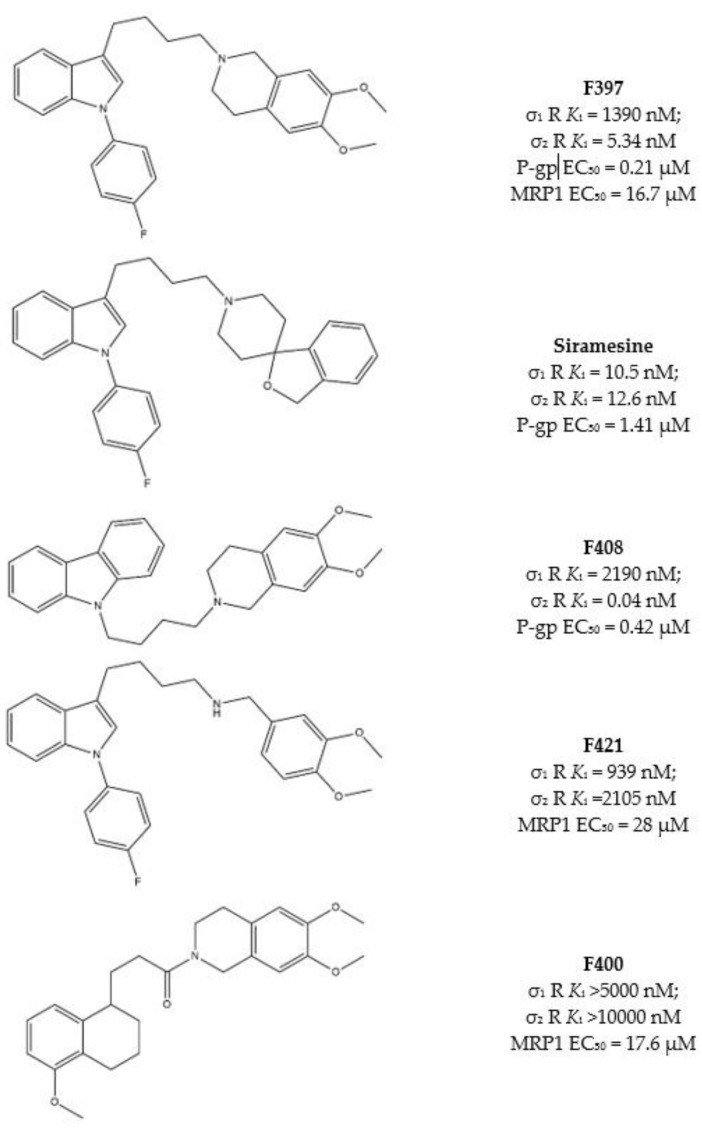
F397, Siramesine, F408, F421 and F400 structures and *K*_i_ values for σ receptors, EC_50_ for P-gp or MRP1.

As some σ receptor ligands are P-gp modulators, Niso and colleagues, studied the ability of some σ_2_ receptor ligands to exert CS, exploiting their interaction with P-gp [46,62,63,89,90]. Ligands were chosen because of the presence of some basic moieties (i.e., 4-(4-fluorophenyl) and 4-cyclohexyl -piperidines or -piperazines and 6,7-dimethoxy-1,2,3,4-tetrahydroisoquinoline) and hydrophobic groups (i.e., carbazole, tetraline, indole and *N*-(4-fluoro-phenyl)indole) with consolidated σ_2_ receptor affinities together with their likely interaction with P-gp. Combination of these basic and hydrophobic moieties led to several high affinity σ_2_ receptor ligands, and the indole-based compounds F397, Siramesine and the carbazole derivative F408 (Figure 5) displayed the most promising dual-target profile because of their affinity/activity at σ_2_ receptor and P-gp. However, CS was only exerted by Siramesine and F408, with a more potent cytotoxicity in MCF7dx (MDR cells) than in the parent MCF7 (non-MDR) cell line. ATP consumption in the same couple of cells (MCF7 and MCF7dx) was evaluated, and while ATP content was lower in MCF7dx than in MCF7 upon F408 and Siramesine administration, the same effect on ATP was not exerted by F397. These data together demonstrated that the CS properties of F408 and Siramesine are due to their activity at P-gp as substrates, with the activation of the futile ATP cycle (to sustain the active efflux of the drug) and increased ROS production. By contrast, F397 was devoid of such an effect because it is a P-gp inhibitor with no ATP consumption and therefore devoid of CS properties mediated by P-gp.

These compounds were investigated in other cell lines couples, such as HT29/HT29dx (colorectal cancer) and A549/A549dx (lung cancer) providing results in accordance with the CS exerted in MCF7 cell lines pairs. Again, F408 and Siramesine demonstrated their CS properties, which were exceptionally important in the HT29 cell lines pair for F408. Indeed, in HT29dx the carbazole derivative F408 produced a 60% cell death at 1 µM concentration. Moreover, studies performed on the mitochondrial respiratory chain revealed that treatment of resistant cells with F408 and Siramesine reduced electron flux and ATP supply, so that these ligands are able to activate multiple cytotoxicity mechanisms [91] and appear as MTDLs worthy to be explored to overcome MDR.

Subsequently, similar studies were performed on the lesser-studied MRP1, which effluxes glutathione (GSH), the tripeptide essential for the correct redox state of cells. Verapamil, the L-type calcium antagonist, can bind MRP1 and stimulate GSH massive extrusion that leads to the activation of apoptotic mechanisms. These properties render Verapamil a collateral sensitizer upon interaction with MRP1 [88].

Structure similarity between Verapamil and some σ_2_ receptor ligands prompted Riganti et al. to screen a library of σ_2_ ligands for their MRP1 activity. Among the most active compounds, the indole-based structures F397 [46] and F421 [90] and the tetralin based amide F400 (Figure 5), [89] were valuable modulators of MRP1 and showed CS in different cells. In particular, F397, F421 and F400 induced CS in MDCK/MDCK-MRP1, A549/A549dx and HT29/HT29dx, and cytotoxicity in MCF7, SKBR3, T74D and MDA-MB-231.

All three compounds were found to deregulate GSH/GSSG ratio and increase ROS production, producing cytotoxicity, especially in MRP1 overexpressing cells.

It is worth noting that F397 did not exert P-gp-mediated CS in MCF7/MCF7dx cells (see above), but emerged as a MRP1-mediated collateral sensitizer in cells where MRP1 is overexpressed. Indeed, MCF7dx cells are devoid of MRP1, so that the data from F397 strongly support the involvement of the P-gp in the CS exerted by F408 and siramesine, while MRP1 is involved in the CS induced by F397, F421 and F400.

These pieces of evidence prompted to further investigate the effect of these σ_2_ receptor ligands in co-administration with cis-Pt, that is one of the clinical antitumor drugs that suffers from MDR. In vitro and in vivo combinations of F397 or F421 with Cis-Pt re-sensitized A549dx cells to cis-Pt and reduced tumor growth without signs of toxicity [92].

**Figure 6:**
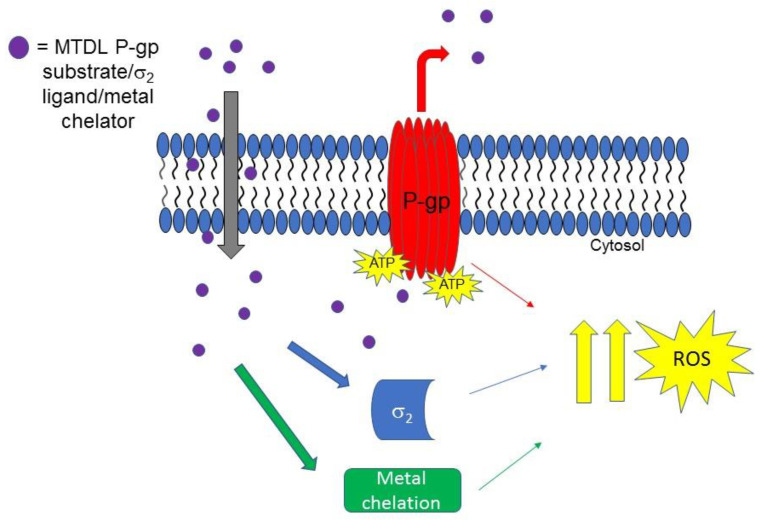
MTDL acting upon interaction with σ_2_ and P-gp proteins and metal chelation. The MTDL, that is a P-gp substrate, upon efflux, activates the futile ATP cycle that leads to ROS increase. The effect is synergized by interaction with σ_2_ receptor and metal ions chelation.

Another step forward on the development of MTDLs based on σ_2_ receptor for the treatment of tumors was taken when novel isatin-β-thiosemicarbazones (IβTs) were produced. IβTs, upon chelation of metals (such as iron and cupper ions) promote ROS production, and have been previously reported as CS inducer [93]. Therefore, according to an MTLDs approach, IβTs core was functionalized with σ_2_ targeting basic moieties with the aim to improve the selectivity for cancer cells that overexpress σ_2_ receptors [94]. In Figure 6, the MTDL strategy targeting σ_2_ receptors P-gp efflux pump and metal chelation is depicted.

**Figure 7:**
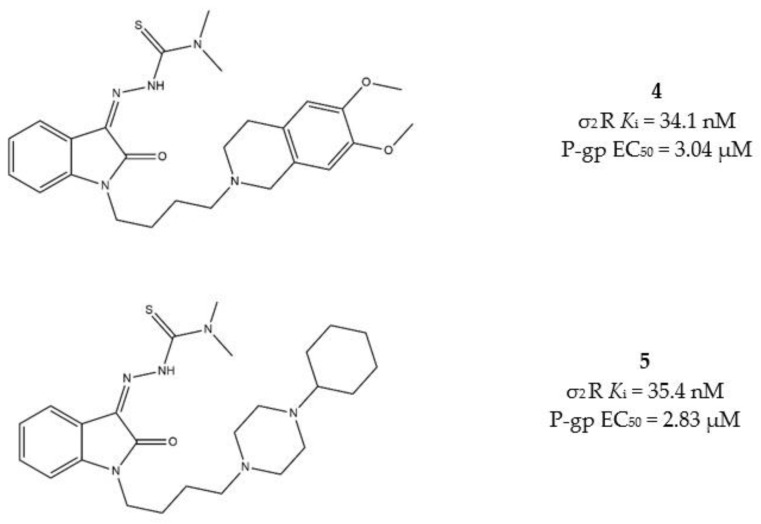
Structures 4 and 5 and their *K*_i_ for σ_2_ receptors and EC_50_ values for P-gp.

The same basic moiety should also determine the interaction with the P-gp. Among the diverse IβTs, the *N,N*-dimethylthiosemicarbazones bearing the 1-cyclohexylpiperazine, or the 6,7-dimethoxytetrahydroisoquinoline as the basic moieties (compounds 4 and 5, Figure 7), provided the best results in terms of cytotoxicity in MCF7/MCF7dx and A549/A549dx, showing important activity also against MDR cells. Therefore, these two compounds underwent a rational deconstruction approach in order to elucidate their structure–activity relationships (SAR) and understand the impact of each target in the overall effect [95]. The activity of the novel compounds was evaluated in pancreatic tumors in vitro, in a panel of human (MIAPaCa-2, BxPC3, AsPC1 and Panc-1) and mouse (Panc02, KP02 and KCKO) cancer cell lines, and in vivo in a murine KP02 tumor model (C57BL/6 mice). This study revealed that the sole *N,N*-dimethylthiosemicarbazone portion is responsible of the cytotoxicity also in the absence of the σ_2_ receptor targeting moiety. Nevertheless, the IβTs devoid of the σ_2_ targeting basic moiety resulted in foci of pulmonary metastases in mice, while the σ_2_ targeting IβTs were equally effective and devoid of side effects. The investigated mechanism of action of σ_2_ receptors targeting compounds showed ROS increase, caspase-3 activation and mitochondria superoxide production [95].

All these data together support the higher selectivity for cancer cells that the σ_2_ targeting exerts. Therefore, according to a multitarget perspective, both *N,N*-dimethyl-thiosemicarbazone and σ_2_ receptors targeting basic moieties are advisable to produce a strong and selective antitumor activity and poor side effects.

## 3. Conclusions

The present review summarizes the progress in the knowledge about σ receptors with a focus on the σ_2_ subtype’s involvement in the oncology field. The role in the proliferative status of cells has directed the σ_2_-related scientific interest towards cancer research. Overall, we have described herein the most promising σ_2_ receptor ligands in the perspective of anticancer therapies, with a focus on the development of σ_2_-based MTDLs ligands. The combination of the σ_2_ targeting moieties, with molecules that activate different apoptotic pathways results in either a synergistic antitumor action or in a targeted delivery to cancers that overexpress the σ_2_ protein. The increasing structural knowledge about this receptor, that has culminated with the disclosure of the crystal structure, together with the successful examples reported herein, may spark novel studies to exploit the σ_2_ subtype as an innovative strategy for the development of MTDLs as anticancer polypharmacological agents, with targeted delivery and improved activity against (resistant) cancers.
